# Comparative analysis of Fenghuang Dancong, Tieguanyin, and Dahongpao teas using headspace solid-phase microextraction coupled with gas chromatography-mass spectrometry and chemometric methods

**DOI:** 10.1371/journal.pone.0276044

**Published:** 2022-10-13

**Authors:** Zhangwei Li

**Affiliations:** Institute of Chemistry and Environment Engineering, Hanshan Normal University, Chaozhou, P. R. China; University of Pisa, ITALY

## Abstract

Fenghuang Dancong, Tieguanyin, and Dahongpao teas are belonged to semi-fermented oolong teas and are famous for their unique aroma. However, reports regarding the systematic comparison, differentiation, and classification of the volatile components of these three types of oolong teas are lacking. In this study, we aimed to establish a method for distinguishing these three types of oolong teas. The volatile components in a total of 21 tea samples of these three types of oolong teas were extracted, determined, and identified by headspace solid-phase microextraction (HS-SPME) combined with gas chromatography-mass spectrometry (GC-MS). In addition, chemometric methods such as hierarchical cluster analysis (HCA), principal component analysis (PCA), and orthogonal partial least squares discriminant analysis (OPLS-DA) were used for distinguishing and classifying the three types of oolong teas on the basis of the similarities and differences in the volatile components. The results showed that 125 volatile components were extracted and identified from the three types of oolong teas, among which 53 volatile components overlapped among the samples. The results of HCA indicated that the samples of each of the three types of oolong teas could be placed in one category when the t value was 220. The results of PCA and OPLS-DA showed that the volatile components such as dehydrolinalool, linalool oxide II, linalool, α-farnesene, linalool oxide I, β-ocimene, nerolidol, cis-3-butyric acid folate, myrcene, and (Z)-hexanoic acid-3-hexenyl ester are the characteristic components, which can be used to distinguish the three types of oolong teas. We developed a simple, fast, and efficient method for distinguishing three types of oolong teas and provided a feasible technique for the identification of oolong tea types.

## Introduction

Tea is one of the most popular drinks in the world owing to its aroma and mellow taste. Tea originated in China, which is currently the world’s main tea producer and consumer [1]. Tea leaves can be divided into non-fermented green tea (Longjing, Biluochun, Huangshan, Maojian), semi-fermented oolong tea (Fenghuang Dancong, Tieguanyin, Dahongpao), and fully fermented tea (Pu’erh tea, Yunnan black tea) based on the degree of fermentation and the production process [2]. Among these, oolong tea is popular in southern China for its unique aroma and soft taste. The main varieties of oolong tea include Fenghuang Dancong tea from Fenghuang Mountain in Chaozhou City, Guangdong Province, Tieguanyin from Anxi County, Fujian Province, and Dahongpao from Wuyi Mountain in the Fujian Province. Fenghuang Dancong tea is famous, especially in the Chaoshan area, for its special and long-lasting flower and fruit and honey fragrance [3]. Tieguanyin has a natural orchid fragrance, pure taste, and long-lasting fragrance and is one of the top ten teas in China. Dahongpao has a rich fragrance and tastes sweet in water. Furthermore, these three types of teas contain beneficial ingredients such as amino acids and vitamins, which are required by the human body. These teas are exported to Japan, South Korea, and Southeast Asia and are well-received by consumers everywhere [4].

As Fenghuang Dancong, Tieguanyin, and Dahongpao are semi-fermented teas, their production processes are similar; as a result, the shapes of the final processed teas are similar, which renders the task of distinguishing these three types of teas on the basis of appearance difficult [4]. Therefore, illegal vendors take advantage of this similarity in appearance and sell low-end teas as high-end teas. This damages the interests of consumers, disrupts the normal order of the tea market, and impacts the economy. Currently, these three types of teas are mainly distinguished on the basis of the sensory review method, which is time-consuming and cumbersome and requires experienced reviewers with strong subjectivity [5]. Therefore, the development of a simple, fast, objective, correct, and reliable analysis method is important for regulating the order of the tea market and protecting the interests of consumers.

Volatile components are characteristic of tea types and are the main source of tea aroma. The aroma of tea mainly depends on the place of origin of the tea, variety of tea, tea tree planting method, growth environment of the tea tree, and the production process of tea. Therefore, the volatile components of tea can be used as the characteristic fingerprint to distinguish and identify the variety, grade, and origin of tea [6]. Researchers often use chemometric tools such as hierarchical cluster analysis (HCA), principal component analysis (PCA), and orthogonal partial least squares discriminant analysis (OPLS-DA) for analyzing the similarities and differences in the volatile components of tea samples, tracing the origin of tea trees, distinguishing tea varieties and tea grades, and determining the characteristic volatile components of tea [7–9]. However, currently, reports on the use of chemometric methods such as HCA, PCA, and OPLS-DA for analyzing the volatile components of Fenghuang Dancong, Tieguanyin, and Dahongpao teas and for determining the characteristic volatile components of these three oolong teas are lacking.

The volatile components of tea should be completely extracted for the accurate determination of their content. The SDE (simultaneous distillation extraction) method is often used to extract the volatile components of tea. In this method, the tea sample and the solvent, usually methylene chloride, are mixed in a closed glass device and heated so that their vapors mix completely, and the method is repeated. This method is time-consuming and cumbersome, and the sample is heated for a long time at a high temperature, which leads to decomposition and oxidation of the volatile components [10]. As a result, the extracted compounds cannot completely reflect the original aroma characteristics [11]. Headspace solid-phase microextraction (HS-SPME) is a solvent-free volatile component extraction method integrating sampling, extraction, concentration, and injection, which has the advantages of being rapid, simple, and solvent-free, and uses low extraction temperature. The extracted volatiles reflect the original aroma characteristics of tea to the highest extent, and this method has been successfully used for extracting various volatile components of tea [12–14].

Previous studies have evaluated the volatile components of three types of oolong teas, including Fenghuang Dancong [3], Tieguanyin [15], and Dahongpao [16], but the number of samples studied was insufficient. Furthermore, reports regarding the systematic comparison, differentiation, and classification of the volatile components of these three types of oolong teas are lacking. For instance, in a previous study [3], we extracted, identified, and analyzed the volatile aroma components of Fenghuang Dancong tea, but we did not compare and analyze these aroma components with those of other varieties of oolong tea, such as Tieguanyin and Dahongpao. This is not suitable for studying the differences between oolong tea varieties and establishing their fingerprints. Therefore, in this study, we aimed to use HS-SPME combined with gas chromatography-mass spectrometry (GC-MS) to extract and identify the volatile components of three types of oolong teas, namely, Fenghuang Dancong, Tieguanyin, and Dahongpao. In addition, chemometric methods, such as HCA, PCA, and OPLS-DA, were used to classify the three oolong teas based on the similarities and differences in the volatile components and to identify the characteristic volatile components in the three oolong teas. Our study provides a feasible technique for the identification of oolong tea types.

## Materials and methods

### Tea samples

Three types of tea samples were used in this study. In total, 21 samples, namely, Fenghuang Dancong tea (7 samples, numbered F1-F7, all purchased from Fenghuang Dancong tea Origin: Fenghuang Town, Chao’an County, Guangdong Province, China), Tieguanyin oolong tea (7 samples, numbered T1-T7, all purchased from Tieguanyin Oolong Tea Origin: Xiping Town, An’xi County, Fujian Province, China), and Dahongpao oolong tea (7 samples, numbered D1-D7, all purchased from DahongPao Oolong Tea Origin: Wuyishan City, Fujian Province, China) were used. All samples were prepared in year 2020. The tea samples were ground, passed through a 40-mesh sieve, and stored in a dry place.

### HS-SPME extraction

3.5 grams of each sample were weighed in a 150 ml sample bottle, followed by the addition of 10 ml ultrapure water and stirring with a magnetic stirrer. The sample was sealed using a sample bottle cap with a PTFE/silica gel septum and incubated in a 65°C water bath for 5 min. Then, a manual sampler equipped with a 65-μm aged PDMS/DVB extraction head was inserted. After 60 min of headspace extraction at 65°C, the sampler was taken out and immediately inserted into the injection port of the GC instrument to desorb for 5 min. At the same time, the instrument was started to collect data [17, 18].

### GC-MS analysis of aroma components

The following GC conditions were used: TG-5MS elastic quartz capillary column (30 m × 0.25 μm × 0.25 μm) (Trace ISQ); inlet temperature, 250°C; carrier gas, high purity helium (purity >99.9999%); flow rate, 1 ml/min; splitless injection. The heating program was as follows: hold at 60°C for 1 min; increase to 230°C at a rate of 10°C·min^-1^; hold for 10 min; increase to 300°C at the rate of 40°C·min^-1^.

The MS conditions were as follows: electron ionization source; ion source temperature, 250°C; electron energy, 70 eV; quadrupole temperature, 150°C; mass scanning range, 40–400 amu.

### Data analysis

The National Institute of Standards and Technology (NIST) library was used to retrieve the data obtained using GC-MS analysis, and the components with matching degree greater than 70 were retained. The retention index (RI) of each component was calculated from its retention time and the retention time of adjacent n-alkanes. Normal alkanes (C8-C40) were purchased from Sigma-Aldrich, USA. The RI of each chromatographic peak was qualitatively compared with the RI in the literature [19, 20]. The peak area normalization method was used for quantitative analysis.

The significant differences between the components of the three teas were analyzed using the SPSS software. The Duncan’s test (p < 0.05 significance level) was conducted to compare study means. HCA, PCA, and OPLS-DA were performed using the SIMCA-14 software.

## Results and discussion

### The results of the GC-MS analysis of the three types of oolong tea volatile components

**Fig 1:**
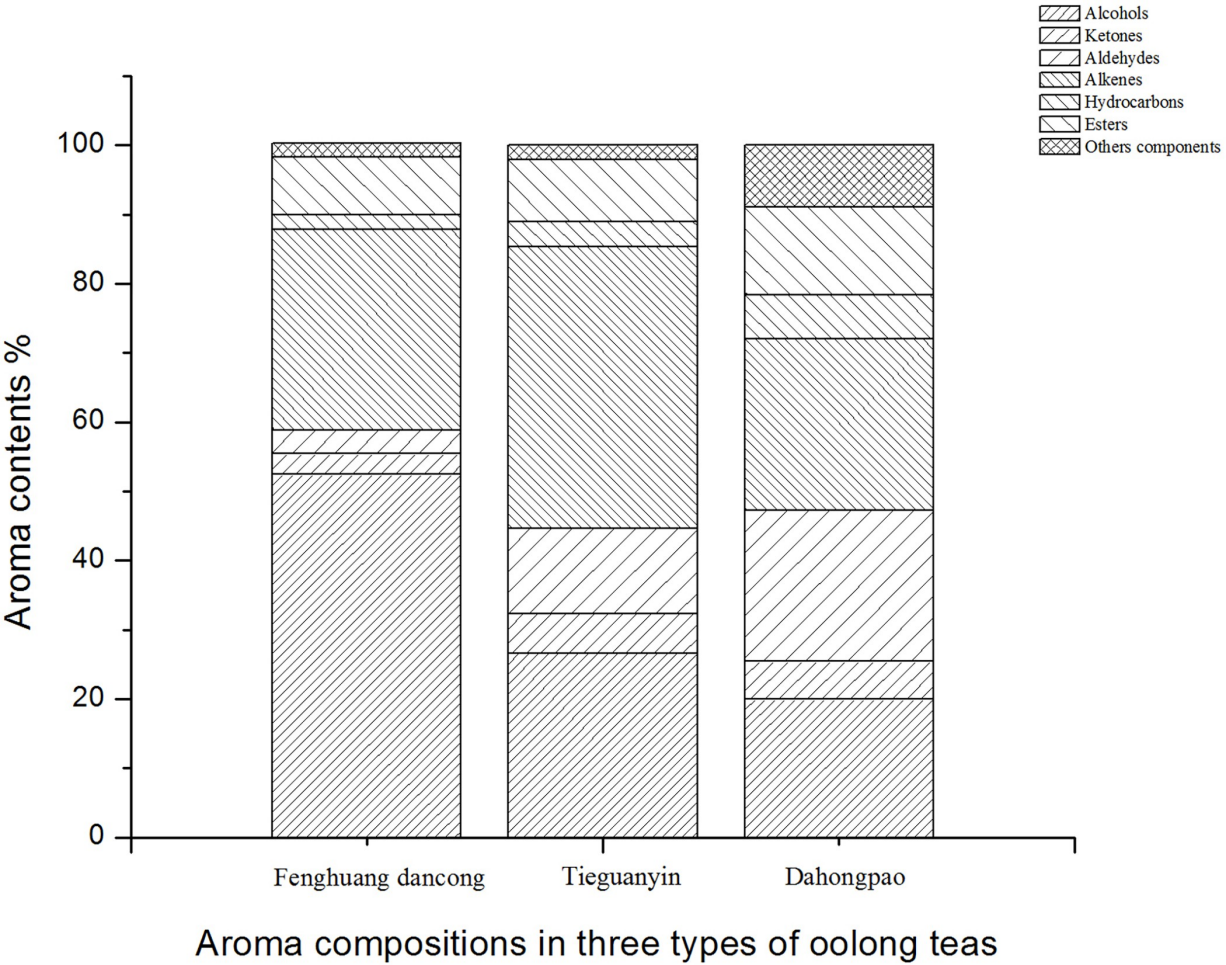
Different chemical classes of volatile compounds and their relative contents found in three types of oolong teas.

The results of the GC-MS analysis of the three types of oolong tea volatile components are shown in Table 1 and Fig 1. In total, 125 volatile aroma components were detected, including 25 alcohols, 25 esters, 24 alkenes, 12 aldehydes, 12 ketones, 13 alkanes, and 14 other components (S1–S21 Figs). Eighty-eight of these components were present in Fenghuang Dancong, 82 in Tieguanyin, and 96 in Dahongpao tea. The three types of oolong teas contained 53 of common ingredients, among which linalool, linalool oxide I, linalool oxide II, nerolidol, and β-ionone were high in content (Table 1). Fig 1 shows the quantity of various alcohols, ketones, esters, aldehydes, alkanes, alkenes, acids, and aromatic hydrocarbons in the volatile aroma of the three types of oolong teas. In tea, linalool and geraniol are both produced by terpenoid synthase from geranyl pyrophosphate (GPP), and linalool is further oxidized to form dehydrolinalool and various other linalool oxides [21]. Linalool presents floral and citrus flavors, with a low odor threshold of only 0.6 μg/L [22]; it is easily captured by the human olfactory system and is an important aroma component of Fenghuang Dancong tea. In a previous study, we detected 1.71–13.98% linalool in many samples of Fenghuang Dancong tea, which was similar to the results of this study [3]. The linalool contents in Tieguanyin and Dahongpao oolong teas were lower than that in Fenghuang Dancong tea, indicating that the high concentration of linalool contributes to the aroma characteristics of the Fenghuang Dancong tea. Dehydrolinalool, associated with the fresh and sweet fragrance of flowers and fruits, is released from linalool glycosidic precursors [11]; its content in the Fenghuang Dancong tea ranges from 16.93% to 29.70%, which considerably affects the aroma of this tea and distinguishes it from other oolong teas. Linalool oxide presents sweet and citrus aromas. In this study, high concentrations of linalool oxides I and II were detected in Fenghuang Dancong and Dahongpao teas. This is similar to the result reported by Li [3] and Zhu [16]. Geraniol has rose fragrance, and its sensory threshold (3.2 μg/L) is higher than that of linalool [22]. The geraniol content was higher in Dahongpao oolong tea than that in the other two oolong teas. The contents of nerolidol, produced from farnesyl pyrophosphate (FPP) by nerolidol synthase [23], are higher in Tieguanyin and Dahongpao teas. This result is similar to that reported by Lin [4] and Zhu [16]. Lin [4] reported that the nerolidol content in Tieguanyin was 12.08%, and it was 4.16% in Dahongpao [16]. In addition, a previous study showed that the nerolidol content in fresh tea was low and that more nerolidol was produced during tea processing [17]. In this study, the alcohol contents of the Fenghuang Dancong, Tieguanyin, and Dahongpao teas reached 52.49%, 26.61%, and 19.94%, respectively; these values are similar to the results of previous studies, but they differ from those detected in Pu’erh tea, green tea, and black tea. This may be one of the differences in the volatile aroma compositions of oolong tea and other teas [24].

**Table 1:** Volatile compounds of Fenghuang Dancong, Tieguanyin, and Dahongpao teas detected using GC-MS.

No.	RI	Compounds	Relative content [%(range)]		
			Fenghuang Dancong (n = 7)	Tieguanying (n = 7)	Dahongpao (n = 7)
1	800	Hexanal	0.00b	0.00b	1.60b (0–4.88)
2	802	2(1H)-Pyridinone	0.33a (0–2.34)	0.18a (0–1.25)	0.00a
3	811	*Trans*-3,4-Epoxynonane	0.00b	0.00b	0.55a (0–1.83)
4	820	Methylpyrazine	0.00b	0.00b	1.36a (1.05–1.79)
5	826	3-Furaldehyde	0.04b (0–0.26)	0.00b	9.71a (7.14–12.18)
6	847	2,5-Cyclooctadien-1-ol	0.00b	0.00b	0.28a (0–0.85)
7	852	2-Hexenal	0.00b	0.00b	1.07a (0–2.33)
8	878	3-Decyn-2-ol	0.00b	0.00b	0.13a (0–0.35)
9	892	1,3,5,7-Cyclooctatetraene	0.00b	0.00b	2.75a (0–3.96)
10	910	2,5-Dimethypyrazine	0.00a	0.00a	0.17a (0–0.65)
11	956	5-Methyl-2furancarboxaldehyde	0.00a	0.12a (0–0.51)	0.00a
12	957	Benzaldehyde	0.00b	0.00b	5.62a (4.67–6.51)
13	959	1-Heptanol	0.31a (0.41–1.38)	0.00a	0.00a
14	973	2,5-Dimethyl-2,4-Hexadiene,	0.00b	0.00b	1.93a (1.67–2.19)
15	986	6-Methyl-5-Hepten-2-one	0.37b (0.08–1.47)	1.28ab (0–3.56)	2.04a (1.21–4.10)
16	988	6-Methyl-5-Hepten-2-ol	0.00a	0.66a (0–3.04)	0.00a
17	992	Myrcene	10.28a (1.92–19.51)	0.00b	0.00b
18	1002	Decane	0.00b	0.00b	4.61a (3.38–7.50)
19	1003	1,7,7-Trimethyl -heptane-2,3-dione	0.18a (0–1.25)	0.80a (0–3.22)	0.00a
20	1015	*α*-Terpinene	0.04b (0–0.29)	0.88b (0–3.86)	5.79a (4.10–8.61)
21	1021	*o*-Cymene	1.27a (0–3.48)	0.00b	0.00b
22	1030	Limonene	1.80a (0–4.75)	0.00b	0.00b
23	1033	Cis-*β*-ocimene	6.72a (1.81–10.60)	0.00b	0.26b (0–1.27)
24	1039	*α*-Pinene	3.46a (0–7.74)	0.00b	0.00b
25	1041	Benzyl alcohol	0.00b	0.57a (0–1.19)	0.12b (0–0.31)
26	1050	Phenylacetaldehyde	3.14b (1.53–5.34)	9.21a (4.31–15. 19)	0.22c (0–0.87)
27	1056	*γ*- Terpinene	3.21b (2.12–4.65)	0.00c	8.96a (7.75–10.74)
28	1062	3-Carene	0.55a (0–1.37)	0.00b	0.00b
29	1073	1-Octanol	0.00a	0.67a (0–3.87)	0.11a (0–0.79)
30	1078	Linalool oxide I	9.16a (4.24–13.31)	0.21c (0–1.45)	6.14b (4.64–7.26)
31	1093	Linalool oxide II	6.45a (4.34–9.54)	0.03c (0–0.22)	2.45b (2.13–2.70)
32	1100	Undecane	0.00a	1.26a (0–8.82)	0.37a (0–2.56)
33	1103	Linalool	8.49a (4.13–12.48)	0.56b (0–1.73)	0.27b (0–1.86)
34	1105	Nonanal	0.00b	1.08a (0–2.29)	0.74ab (0–2.57)
35	1108	Dehydrolinalool	22.36a (16.93–29.70)	0.00b	0.00b
36	1118	Dodecanal	0.00b	1.22a (0.85–1.84)	0.92a (0–1.75)
37	1134	*Cis*-Mentha-2,8-dien-1-ol	0.72a (0–2.00)	0.01b (0–0.05)	0.01b (0–0.08)
38	1145	Benzonitrile	0.26b (0–0.99)	0.13b (0–0.35)	1.29a (0–2.03)
39	1156	4-Isopropylcyclohexanone	0.08a (0–0.56)	0.00a	0.00a
40	1163	1-Nonanol	0.06b (0–0.45)	0.06b (0–0.32)	0.84a (0–1.42)
41	1171	Naphthalene	0.00b	0.00b	0.87a (0–1.13)
42	1176	Epoxylinalool	0.34a (0–2.36)	0.09a (0.16–0.28)	0.00a
43	1183	Methyl4-methylphenylacetate	0.00b	0.00b	0.83a (0.70–0.99)
44	1187	*Cis*-3-Hexenyl butyrate	0.00b	0.65a (0.35–1.26)	0.00b
45	1191	3,4-Dimethyl-o-phenylenediamine	0.20b (0–1.40)	0.00b	2.35a (2.04–2.95)
46	1197	*α*-Terpineol	2.03a (0–4.09)	0.42b (0–2.53)	0.00b
47	1202	Methyl salicylate	0.00b	0.00b	3.57a (2.37–4.77)
48	1207	Decanal	0.20a (0–0.86)	0.25a (0–0.82)	0.14a (0–0.70)
49	1223	*p*-Cyclocitral	0.00b	0.14a (0–0.36)	0.00b
50	1229	Hexyl pivalate	0.41a (0–1.16)	0.43a (0.08–1.55)	0.84a (0.62–1.15)
51	1233	Isovaleric acid-cis-3-hexenyl ester	0.00b	0.16a (0–0.35)	0.00b
52	1236	Nerol	0.17a (0–0.81)	0.00a	0.27a (0–0.56)
53	1245	3,7,7-Trimethyl -hept-2-ene	0.13a (0–0.61)	0.06a (0–0.43)	0.12a (0–0.32)
54	1249	Isoamyl 3-methylvalerate	0.00a	0.02a (0–0.08)	0.00a
55	1257	*β*- Pinene	0.74a (0–5.20)	0.00a	0.00a
56	1263	Geraniol	0.00b	0.24b (0–0.88)	1.03a (0.86–1.16)
57	1273	*cis*-Mentha-1,8-dien-2-ol	0.30ab (0–1.81)	0.07b (0–0.49)	0.66a (0.47–0.74)
58	1280	Nonanoic acid	0.04b (0–0.25)	0.21a (0–0.41)	0.00b
59	1284	3-Cyclohex-1-enyl-prop-2-enal	0.00b	0.00b	0.22a (0–0.57)
60	1289	1-Methylnaphthalene	0.36b (0–0.56)	0.00c	1.01a (0.69–1.40)
61	1296	Pentyl hexanoate	0.00b	0.10b (0–0.69)	0.68a (0.49–0.83)
62	1303	Indole	0.20b (0–0.79)	1.39a (0.66–2.20)	0.45b (0–0.72)
63	1319	*(E*,*E)*-2,4-Decadienal	0.01b (0–0.07)	0.34b (0–1.41)	1.82a (1.07–2.45)
64	1323	Methyl geranate	0.42a (0–1.23)	0.06a (0–0.42)	0.24a (0–0.83)
65	1351	α-Ionene	0.04a (0–0.26)	0.00a	0.04a (0–0.17)
66	1357	4-(2,2-Dimethyl-6-methylenecyclohexyl)butanal	0.02a (0–0.12)	0.01a (0–0.04)	0.03a (0–0.18)
67	1363	1,1,5-Trimethyl-1,2-dihydronaphthalene	0.15b (0–0.71)	0.00b	0.93a (0–1.49)
68	1365	2-Naphthylethanol	0.00a	0.07a (0–0.49)	0.13a (0–0.66)
69	1381	*(Z)*-Hexanoic acid, 3-hexenyl ester	0.11c (0–0.36)	1.64a (0.84–3.10)	0.84b (0.62–0.96)
70	1387	Hexyl hexanoate	0.29b (0–1.15)	0.37b (0–0.84)	1.35a (1.04–1.65)
71	1389	*(E)*- Hexanoic acid, 2-hexenyl ester	0.00a	0.09a (0–0.34)	0.00a
72	1392	*Cis*-Jasmone	0.03a (0–0.22)	0.00a	0.00a
73	1398	Tetradecane	0.13a (0–0.62)	0.20a (0–1.03)	0.26a (0–0.67)
74	1406	*α*-Cedrene	0.64a (0–1.83)	0.00b	0.24ab (0–0.38)
75	1417	Caryophyllene	0.02b (0–0.14)	0.00b	0.22a (0–0.39)
76	1427	*α*-Ionone	0.02a (0–0.15)	0.00a	0.02a (0–0.13)
77	1435	2-Methylbutyl benzoate	0.17ab (0–0.54)	0.10b (0–0.68)	0.39a (0.24–0.55)
78	1440	*(E)*-P-Famesene	0.31a (0–0.81)	0.00b	0.48a (0.33–0.61)
79	1447	Geranylacetone	0.00b	0.03b (0–0.20)	0.10a (0–0.15)
80	1452	1,6,6-Trimethyl-7-(3-oxobut-1-enyl)-3,8-dioxacyclo[5.1.0.0(2,4)]octyl-5-one	0.05a (0–0.34)	0.00a	0.02a (0–0.12)
81	1455	*(E)*-Geranyl acetone	0.29b (0–1.38)	0.83ab (0–3.67)	1.32a (0.99–1.62)
82	1459	*(E)-β*-Farnesene	0.00a	0.02a (0–0.12)	0.05a (0–0.35)
83	1468	*γ*-Cadinene	0.10a (0–0.70)	0.50a (0–1.91)	0.07a (0–0.24)
84	1488	*β*-Bisabolene	0.06a (0–0.45)	0.10a (0–0.69)	0.06a (0–0.45)
85	1494	*Trans-β*-Ionone	1.20a (0–2.74)	2.09a (0.52–4.10)	1.87a (1.10–2.61)
86	1501	Pentadecane	0.00b	0.00b	0.33a (0.12–0.71)
87	1510	*α*-Farnesene	0.14b (0–0.41)	38.03a (19.14–47.63)	2.26b (0.91–8.56)
88	1514	Butylated hydroxytoluene	0.00b	0.00b	0.15a (0–0.41)
89	1527	Dihydroactinidiolide	0.01a (0–0.09)	0.00a	0.03a (0–0.19)
90	1533	Citronella-1,4-diene	0.04a (0–0.27)	0.17a (0–0.50)	0.19a (0–0.47)
91	1557	Geranyl geraniol	0.00a	0.04a (0–0.30)	0.11a (0–0.75)
92	1568	Nerolidol	0.71b (0–1.98)	22.33a (8.31–32.34)	6.08b (3.04–11.28)
93	1579	*Cis*-3-Hexenyl benzoate	0.14b (0–0.39)	0.89a (0–1.46)	0.60a (0.36–0.99)
94	1581	1-Hexadecene	0.00b	0.47a (0–1.49)	0.02b (0–0.14)
95	1589	*Cis*-1-Chloro-9-octadecene	0.01a (0–0.09)	0.03a (0–0.21)	0.06a (0–0.21)
96	1597	1-Octadecyl sulfonyl chloride	0.36a (0–0.92)	0.32a (0–1.10)	0.27a (0–0.48)
97	1599	Hexadecane	0.00b	0.54a (0–1.06)	0.00b
98	1621	Phenol	0.03a (0–0.24)	0.03a (0–0.21)	0.04a0-0.29)
99	1647	*β*-Citrulene	0.02a (0–0.16)	0.03a (0–0.22)	0.00a
100	1656	Tributyl phosphate	0.02a (0–0.13)	0.06a (0–0.39)	0.02a (0–0.15)
101	1668	*β*-Acorenol	0.05ab (0–0.35)	0.00b	0.26a (0–0.70)
102	1696	Sulfurous acid, hexyl pentadecyl ester	0.45a (0–0.75)	0.42a (0–87)	0.20a (0–0.50)
103	1702	Heptadecane	0.03b (0–0.22)	0.37a (0–1.03)	0.03b (0–0.13)
104	1796	Octadecane	0.50ab (0.15–0.81)	0.53a (0–1.23)	0.20b (0.10–0.31)
105	1806	Tert-hexadecyl mercaptan	0.17a (0–0.45)	0.17a (0–0.68)	0.05a (0–0.21)
106	1837	Neophytadiene	0.71ab (0.19–1.38)	0.31b (0–1.08)	1.01a (0.68–1.70)
107	1845	Methyl pentadecanoate	1.02a (0.30–2.20)	0.48a (0–1.79)	0.80a (0.41–1.28)
108	1862	Chlorophyll	0.50ab (0–1.09)	0.23b (0–0.94)	0.69a (0.37–1.03)
109	1875	Octadecyl isobutyl phthalate	0.08a (0–0.53)	0.03a (0–0.24)	0.00a
110	1881	*(Z)*-9-Tetradecen-l-ol acetate	0.43ab (0–0.97)	0.15b (0–0.58)	0.62a (0.40–0.80)
111	1896	Famesyl acetone	0.43a (0–0.80)	0.50a (0.14–0.90)	0.14b (0.11–0.31)
112	1903	Nonadecane	0.00b	0.00b	0.04a (0–0.10)
113	1914	N- [4-bromo-n-butyl]-2-piperidone	0.01a (0–0.01)	0.00a	0.00a
114	1924	Methylhexadecanoate	1.85a (0.19–4.32)	0.65b (0–3.10)	0.35b (0–0.70)
115	1947	Isophytol	0.39a (0–0.99)	0.17a (0–0.64)	0.31a (0–0.48)
116	1970	Octadecyl isobutyl phthalate	0.27a (0–1.80)	0.08a (0–0.53)	0.09a (0–0.20)
117	1995	Ethyl palmitate	0.64a (0.12–1.40)	0.77a (0.11–1.92)	0.15b (0–0.33)
118	2096	9,12-Octadecadienoic acid methyl ester	0.73a (0.17–1.34)	0.56ab (0.26–0.98)	0.26b (0.18–0.35)
119	2103	Methyl 8,11,14-heptadecatrienoate	1.00a (0–2.52)	0.51a (0–1.91)	0.60a (0.29–1.90)
120	2131	Linoleic acid	0.43a (0–1.85)	0.07a (0–0.46)	0.00a
121	2151	3,7,11,15-Tetramethyl-2-hexadecen-1-ol	0.28a (0–1.47)	0.02a (0–0.11)	0.03a (0–0.19)
122	2194	Octadecyl acetate	0.27a (0–0.85)	0.71a (0–3.46)	0.16a (0–0.99)
123	2294	Tridecane	0.13a (0–0.50)	0.18a (0–0.93)	0.01a (0–0.07)
124	2394	Tetradecane	0.03a (0–0.24)	0.10a (0–0.52)	0.00a
125	2494	Pentadecane	0.03a (0–0.18)	0.47a (0–2.77)	0.00a

The same letter in the same row indicates no significant difference (*P* < 0.05) according to Duncan’s test.

Both benzyl alcohol and benzaldehyde are produced from phenylalanine via cinnamic acid by specific enzymes [25]. Benzyl alcohol has a fruity sweet taste, while benzaldehyde has a special almond taste. The benzaldehyde content is high in Dahongpao, and it is an important volatile component of this tea. The benzyl alcohol content in Tieguanyin is higher than that in the other two teas, and the aroma of benzyl alcohol is one of the characteristic aromas that distinguish Tieguanyin from the other two oolong teas. Phenylacetaldehyde is also a volatile aroma substance produced by phenylalanine. In this study, high levels of phenylacetaldehyde were detected in both Tieguanyin and Fenghuang Dancong teas. Phenylacetaldehyde has a honey-like aroma and is often detected in oolong tea [26]. We detected high content of 3-furfural in Dahongpao, and furfural substances are often detected in oolong tea. As furfural is mainly generated during the processing of tea [19], the processing conditions of Dahongpao tea may contribute to its high furfural content [16]. In this study, the contents of aldehyde in Fenghuang Dancong, Tieguanyin, and Dahongpao teas were 3.41%, 12.36%, and 21.87%, respectively, indicating that these teas contained high levels of aldehydes.

Indole in tea is produced from tryptophan. Studies have shown that indole is mainly produced during the fermentation of tea and that it imparts a certain floral fragrance to the tea [27]. In this study, Tieguanyin and Dahongpao oolong teas contained a certain amount of indole, indicating that indole affected the aroma composition of these two oolong teas. Lin [4] found that the indole content in oolong tea ranged from 4.49% to 12.03%. Ma [17] confirmed that indole was mainly produced during the fermentation process. The content of indole in oolong tea after fermentation is 70 times more than that before fermentation. We speculated that the large amount of indole produced during the fermentation of tea is related to the regulation of injury stress during the tea production process [17].

β-Ionone is produced from β-carotene by a carotenoid cleavage enzyme. It has the fragrance of violet flowers; its odor threshold is only 0.2 μg/L, which is easily detected by the human olfactory system, and it contributes considerably to the aroma of tea [28]. In this study, it was found that the three types of oolong teas have high contents of β-ionone, indicating that its aroma is one of the characteristic aromas of oolong teas. Some sesqui-terpenes, such as myrcene, α-pinene, limonene, β-ocimene, γ-terpinene, and α-farnesene, were also detected. Myrcene has a light balsamic aroma; a high content of myrcene was found in Fenghuang Dancong tea, but it was not detected in the other two oolong teas. Shi [29] detected high levels of myrcene in 15 samples of Fenghuang Dancong tea, and its contents ranged from 1.42% to 9.86%, which was similar to the results of this study. Limonene has a refreshing lemon scent, which imparts a faint lemon aroma to tea. We detected limonene in Fenghuang Dancong tea, and its aroma may be one of the characteristic aromas of this tea. Limonene is also an important component of green tea. Some studies have found that the higher the content of limonene in green tea, the higher the grade of the tea [30]. β-Ocimene has a sweet fragrance, while α-pinene is associated with turpentine odor. The contents of these two olefin volatile aroma components were higher in Fenghuang Dancong tea than those in the other two oolong teas, suggesting that these two aromas contribute the most to the characteristic aroma of Fenghuang Dancong tea. In the previous study, we also detected β-ocimene and α-pinene in Fenghuang Dancong tea, which implied that these two volatile aroma substances are the characteristic substances in this tea [3]. Similar to nerolidol, α-farnesene is generated from pyruvate. Initially, pyruvate produces farnesyl pyrophosphate (FPP) via the mevalonate pathway, following which FPP is enzymatically converted to α-farnesene [11]. Ma [17] demonstrated that fresh tea contained low levels of α-farnesene and that a large amount of α-farnesene was produced during the tea making process, which may be related to the mechanism via which tea copes with environmental stress (abiotic stress). In this study, Tieguanyin and Dahongpao were found to have higher α-farnesene contents than Fenghuang Dancong tea. Lin [4] also found a large amount of α-farnesene in oolong teas, which was consistent with the results of this study. γ-Terpinene, with citrus and lemon aroma, is present in high levels in Dahongpao and Fenghuang Dancong teas.

Some esters such as methyl geranate, methyl salicylate, hexyl hexanoate, and ethyl palmitate were also detected in this study. Methyl salicylate has a strong holly oil herbal fragrance. Wang [31] analyzed the aroma substances of 56 types of green tea, oolong tea, and dark tea, and they inferred that methyl salicylate is one of the characteristic aroma substances that distinguish semi-fermented and completely fermented teas. In this study, methyl salicylate was found only in Dahongpao tea but not in the other two oolong teas. This may be related to the production methods of various types of oolong teas. Methyl geranate, with a faint lavender fragrance, was found in all the three types of oolong teas. Hexyl hexanoate appeared to have a fresh and fruity fragrance, and its content was higher in Tieguanyin and Dahongpao teas than that in Fenghuang Dancong tea. Other esters, such as ethyl palmitate, methyl 9,12-octadecadienoate, and methyl 8,11,14-heptadecosatrienoate, were found in all the three types of oolong teas. These esters, formed via dehydration and condensation of higher fatty acids and lower alcohols, have lighter fragrance and contribute less to the aroma of tea [32].

### Chemometric analysis of the three types of oolong tea volatile components

**Fig 2:**
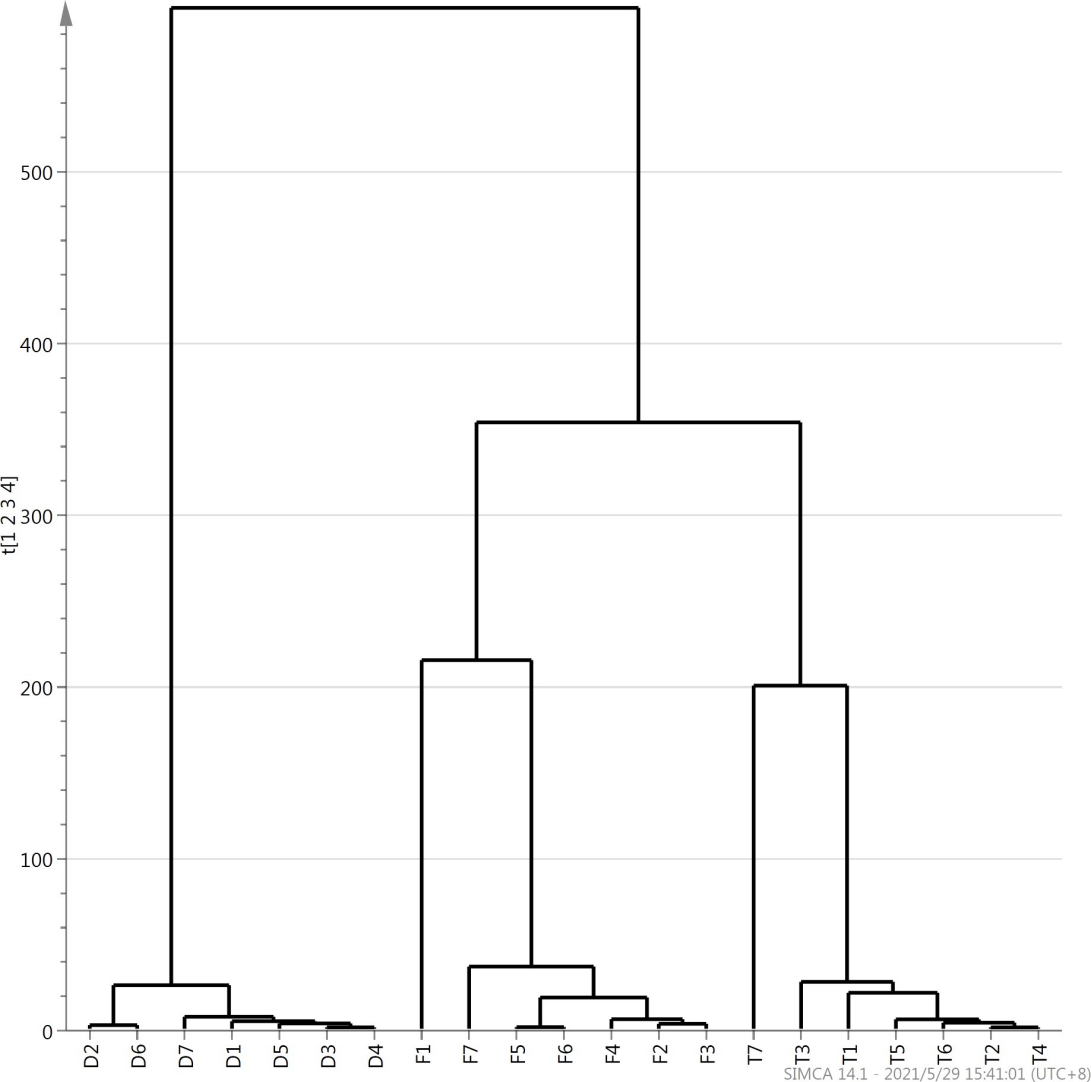
Hierarchical cluster analysis of three types of Fenghuang Dancong, Tieguanyin, and Dahongpao teas. F1-F7: Samples of Fenghuang Dancong; T1-T7: Samples of Tieguanyin; D1-D7: Samples of Dahongpao tea.

HCA is a multivariate statistical method in which samples are gradually aggregated according to the similarity of their quality characteristics. HCA is widely used in distinguishing tea varieties, investigating tea-producing areas, and identifying tea quality [12]. Wang [24] used HCA to analyze two types of dark tea: Pu’erh tea produced in the Yunnan Province and Fuzhuan tea produced in the Hunan Province, and they found that these two types of teas can be clearly distinguished on the basis of their volatile aroma characteristics. In this study, the contents of various volatile components in 21 samples of the three types of oolong teas were used as variables, and the square of Euclidean distance was used as the measurement standard; HCA was performed using the SIMCA 14 software (Fig 2). The clustering results revealed that the three types of oolong teas can be divided into three categories, each consisting of seven samples of each of the three types, when the distance is 220. This indicated that the three types of oolong teas can be distinguished on the basis of the types and contents of the volatile components in the teas.

**Fig 3:**
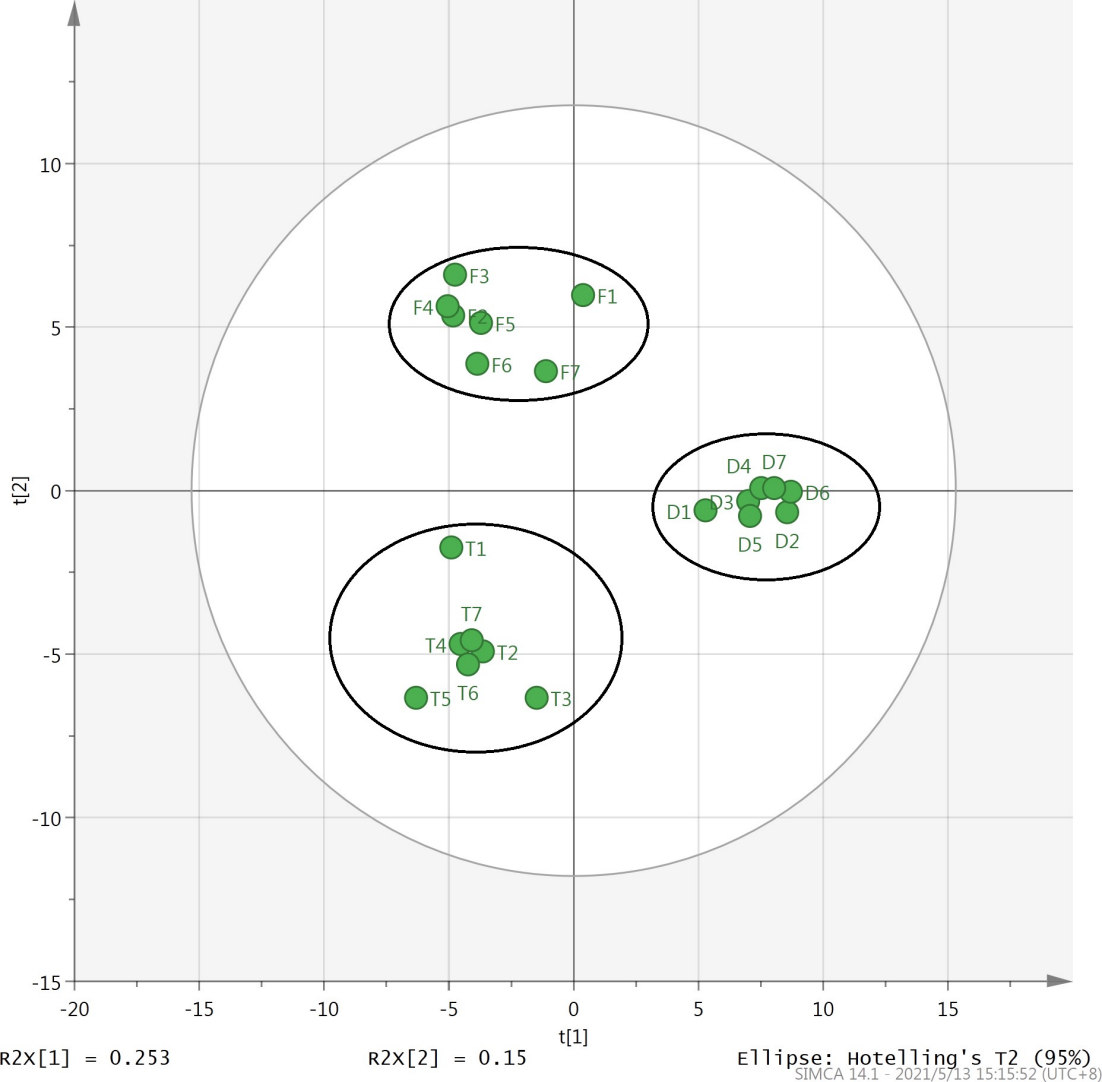
Principal component analysis of Fenghuang Dancong, Tieguanyin, and Dahongpao teas. F1-F7: Samples of Fenghuang Dancong; T1-T7: Samples of Tieguanyin; D1-D7: Samples of Dahongpao tea.

**Fig 4:**
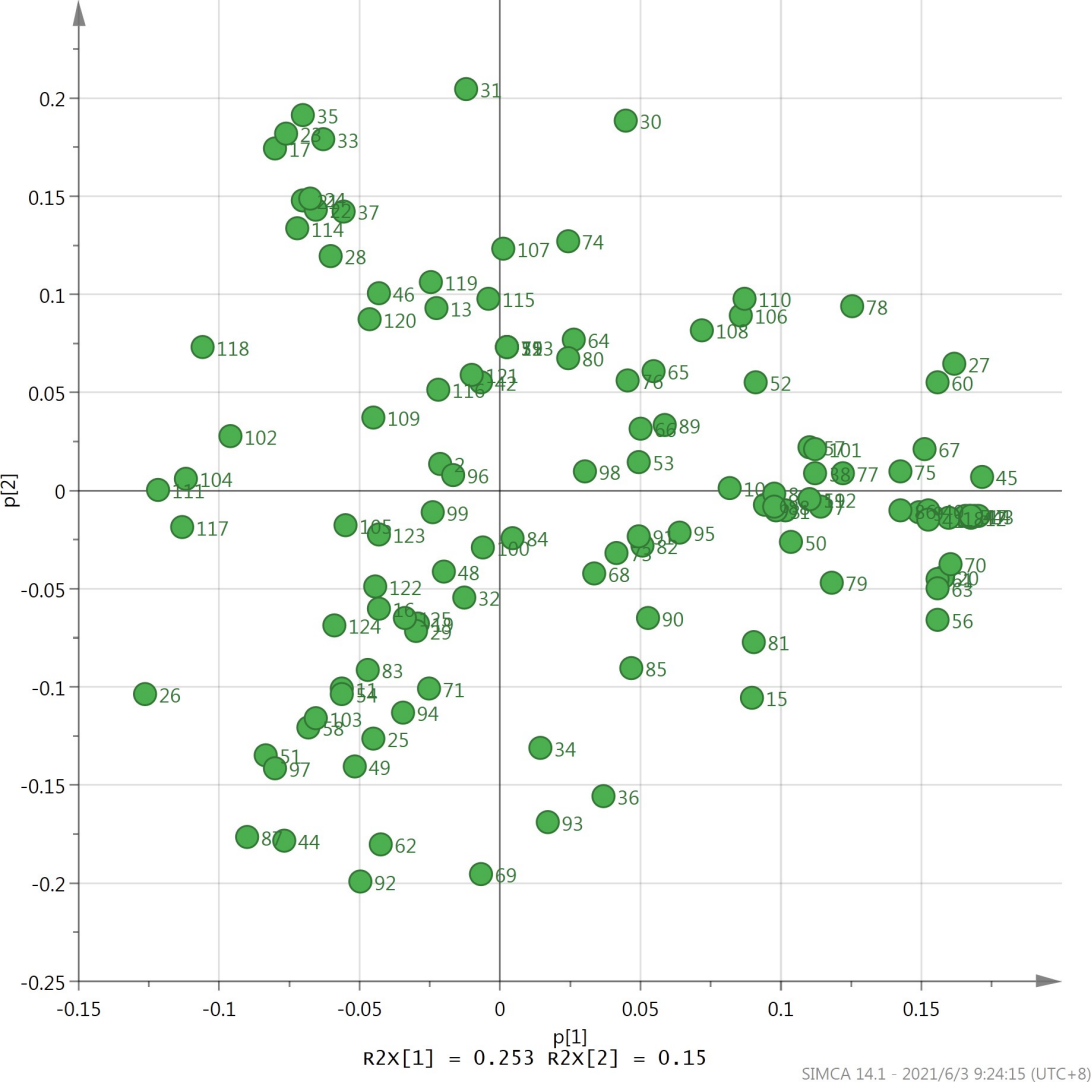
Principal component analysis of the 125 aroma compounds of Fenghuang Dancong, Tieguanyin, and Dahongpao teas. The number of samples in the figure corresponds to the number of volatile compounds reported in Table 1.

PCA is a multivariate statistical method that converts multiple indicators into few unrelated comprehensive indicators and classifies the comprehensive indicators according to certain rules. This analysis can reduce the indicator dimension, condense indicator information, simplify complex problems, and make problem analysis more intuitive and effective. PCA has been used to study the volatile components in tea. Wu [33] used PCA to categorize the Pu’erh green tea obtained from the two production areas into two groups based on its volatile aroma components. The score scatter plot reveals the similarities and differences in the tea samples on the basis of the differences in the types and contents of the volatile components in these samples. The loading scatter plot shows the volatile components that can best distinguish the tea samples. The score scatter plot indicated that the first two principal components (PC1 and PC2) represent 40.3% of the total variability. Although the contribution of PC1 and PC2 is not very high, seven samples each of the Fenghuang Dancong, Tieguanyin, and Dahongpao oolong teas can be distinguished well on PC1 and PC2 (Fig 3). The seven samples of each of the three types of oolong teas can be grouped into one group, which is similar to the result of HCA. The loading scatter plot showed that methyl 4-methyl phenyl acetate, 3,4-Dimethyl-o-phenylenediamine, methyl salicylate, decane, hexanoic acid hexylester, 3-furfural, geraniol, (E,E)-2,4-decadienal, and pentyl hexanoate have higher positive values on PC1, reflecting the difference in the types and contents of the volatile components of Dahongpao and the other two oolong teas. Linalool oxide I, linalool oxide II, linalool, dehydrolinalool, β-ocimene, and myrcene have higher positive values on PC2, indicating the difference on content and type of volatile aroma compounds in the Fenghuang Dancong tea and the other two oolong teas. α-farnesene, cis-3-hexenyl butyrate, indole, nerolidol, and (Z)-hexanoic acid-3-hexenyl ester have higher negative values on PC2, indicating the difference between Tieguanyin and the other two types of oolong teas in terms of the content of volatile aroma compounds (Fig 4). Thus, the PCA method reveals the characteristic volatile aroma components of the three types of oolong teas. The results of PCA corroborate the significant differences in the volatile components of the three oolong tea types shown in Table 1; this confirms that by using chemometric methods such as HCA and PCA, the quality of the three oolong teas can be evaluated based on the types and contents of the volatile components extracted and analyzed using HS-SPME combined with GC-MS.

**Fig 5:**
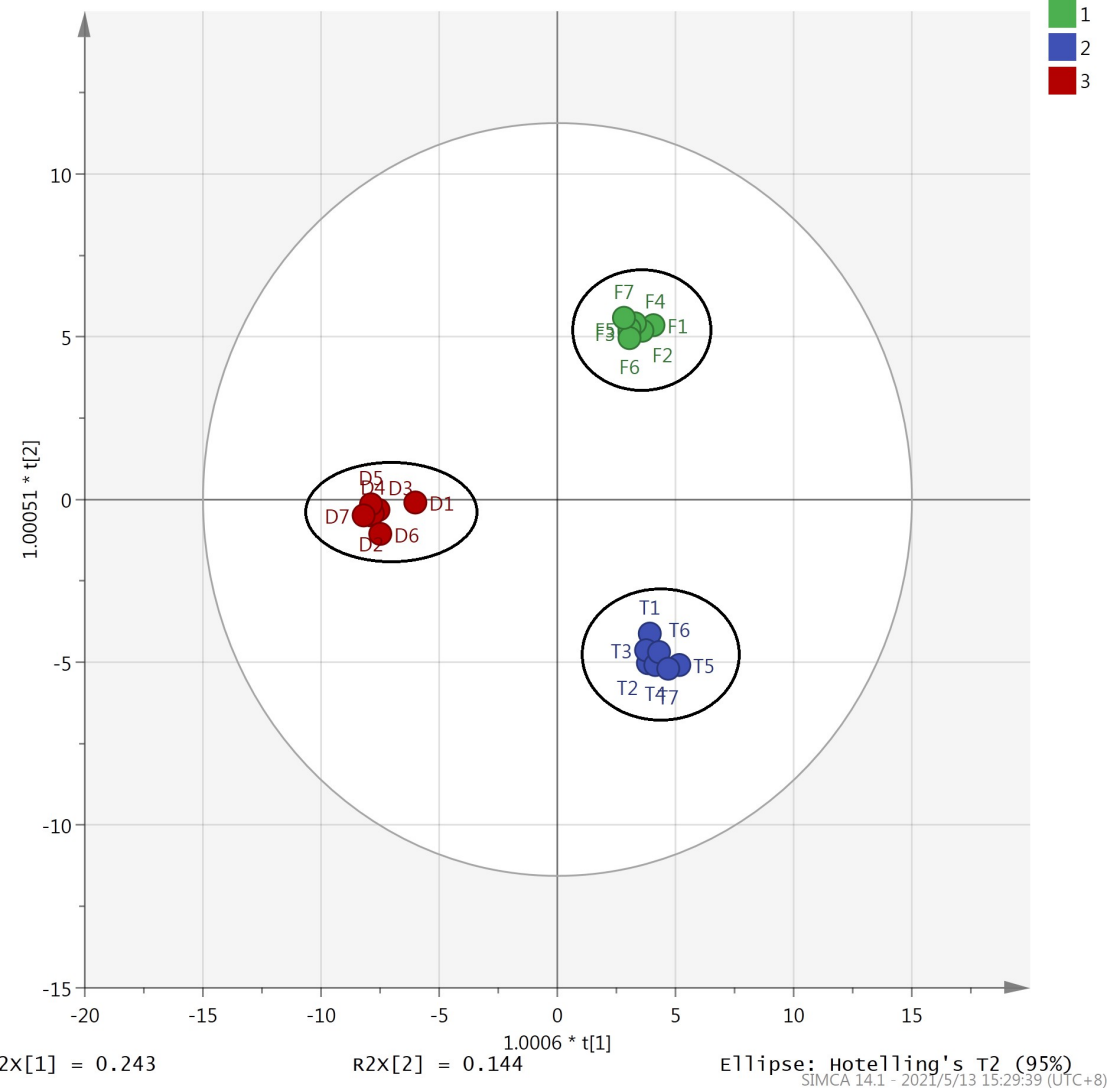
Results of orthogonal partial least squares discriminant analysis of Fenghuang Dancong, Tieguanyin, and Dahongpao teas. F1-F7: Samples of Fenghuang Dancong; T1-T7: Samples of Tieguanyin; D1-D7: Samples of Dahongpao tea.

**Fig 6:**
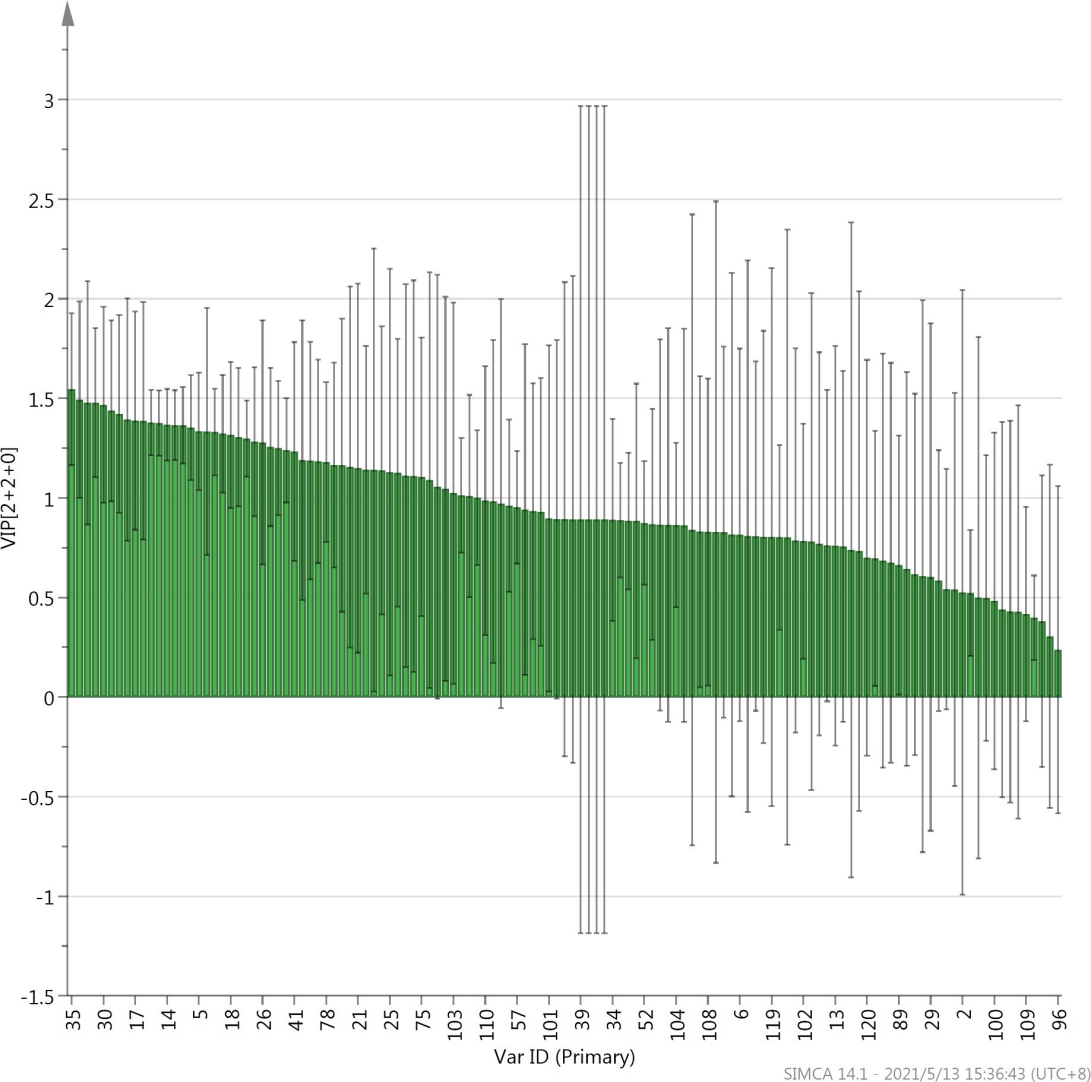
VIP values of the 125 aroma compounds of Fenghuang Dancong, Tieguanyin, and Dahongpao teas. The number of samples in the figure corresponds to the number of volatile compounds reported in Table 1.

OPLS-DA is a supervised statistical method for discriminant analysis. This method uses partial least squares regression to establish the relationship between the types and contents of volatile components in the three types of tea samples [34]. In this study, based on the differences in the types and contents of the volatile aroma components in the three types of oolong teas, the OPLS-DA method was used to investigate the similarities and differences between the 21 samples of the three oolong teas (Fig 5). The score scatter plot shows that similar to the results of PCA, the three types of oolong teas can be divided into three categories, each consisting of seven samples of each of the three types. VIP (variable importance in projection) is the variable weight value of the OPLS-DA model variables, which can be used to determine the impact and explanatory power of the differences in the types and contents of various volatile components for the classification of the three teas. VIP ≥ 1 is a common screening criterion for volatile components. In this study, the volatile aroma components in the three types of oolong teas can be arranged in descending order of type and content as follows: dehydrolinalool, linalool oxide II, linalool, α-farnesene, linalool oxide II, β-ocimene, nerolidol, and myrcene. This suggests that the contents of these volatile aroma components differed considerably in the three types of oolong teas (Fig 6).

In this study, after conducting extraction using HS-SPME, identification via GC-MS, and analysis of the aromas of the Fenghuang Dancong, Tieguanyin, and Dahongpao teas using chemometric methods (HCA, PCA, and OPLS-DA), the characteristic aromas of the three oolong teas were identified. Moreover, we established a method for distinguishing and identifying the three kinds of oolong teas by characteristic aroma components, so as to provide a feasible technique for the identification of oolong tea varieties.

## Conclusions

We reported for the first time a method in which volatile components can be used to distinguish three types of oolong teas of different varieties and origins (Fenghuang Dancong, Tieguanyin, and Dahongpao). The results showed that HS-SPME combined with GC-MS can be used to effectively extract and identify the volatile aroma components of the three types of oolong teas. Furthermore, the combination of chemometric methods such as HCA, PCA, and OPLS-DA can effectively distinguish the three types of oolong teas. In addition, the results showed that dehydrolinalool, linalool oxide II, linalool, α-farnesene, linalool oxide I, β-ocimene, nerolidol, cis-3-hexenyl butyrate, myrcene, and (Z)-hexanoic acid-3-hexenyl ester are the characteristic volatiles that distinguish the three types of oolong teas. Overall, HS-SPME combined with GC-MS and HCA, PCA, and OPLS-DA can effectively distinguish oolong teas of different origins and varieties.

## Supporting information

S1 Fig GC-MS picture of F1 sample. (TIF)

S2 Fig GC-MS picture of F2 sample. (TIF)

S3 Fig GC-MS picture of F3 sample. (TIF)

S4 Fig GC-MS picture of F4 sample. (TIF)

S5 Fig GC-MS picture of F5 sample. (TIF)

S6 Fig GC-MS picture of F6 sample. (TIF)

S7 Fig GC-MS picture of F7 sample. (TIF)

S8 Fig GC-MS picture of T1 sample. (TIF)

S9 Fig GC-MS picture of T2 sample. (TIF)

S10 Fig GC-MS picture of T3 sample. (TIF)

S11 Fig GC-MS picture of T4 sample. (TIF)

S12 Fig GC-MS picture of T5 sample. (TIF)

S13 Fig GC-MS picture of T6 sample. (TIF)

S14 Fig GC-MS picture of T7 sample. (TIF)

S15 Fig GC-MS picture of D1 sample. (TIF)

S16 Fig GC-MS picture of D2 sample. (TIF)

S17 Fig GC-MS picture of D3 sample. (TIF)

S18 Fig GC-MS picture of D4 sample. (TIF)

S19 Fig GC-MS picture of D5 sample. (TIF)

S20 Fig GC-MS picture of D6 sample. (TIF)

S21 Fig GC-MS picture of D7 sample. (TIF)
