# Supplementary figures and images for: Comparative analysis of Fenghuang Dancong, Tieguanyin, and Dahongpao teas using headspace solid-phase microextraction coupled with gas chromatography-mass spectrometry and chemometric methods
Source: PLoS One. 2022 Oct 13;17(10):e0276044. doi: 10.1371/journal.pone.0276044 (PMC9560621; doi:10.1371/journal.pone.0276044)

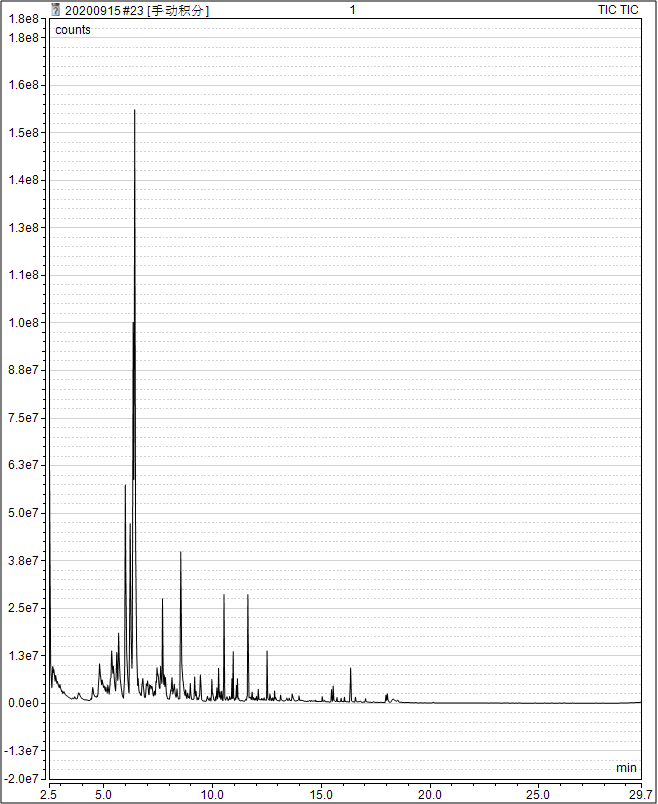

Supplement: S1 Fig — (TIF) [file pone.0276044.s001.tif]

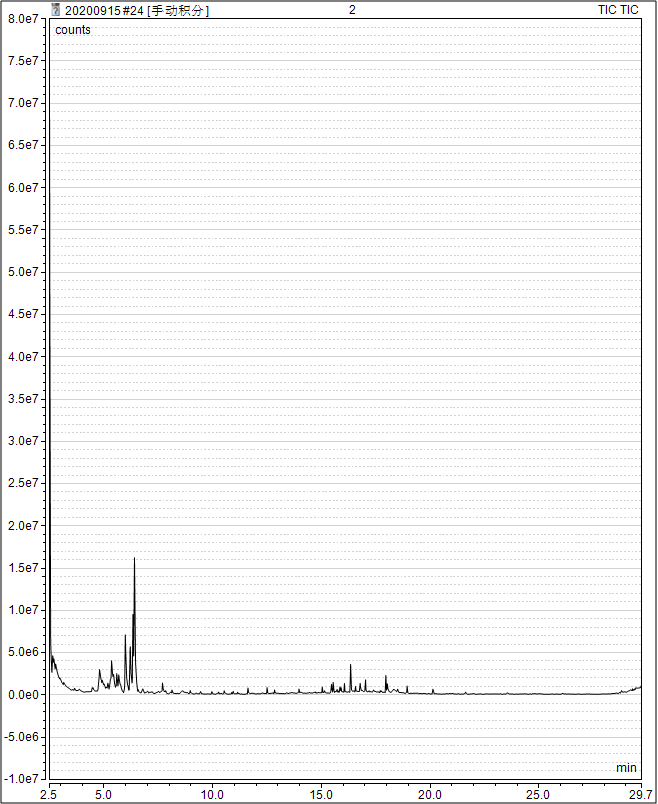

Supplement: S2 Fig — (TIF) [file pone.0276044.s002.tif]

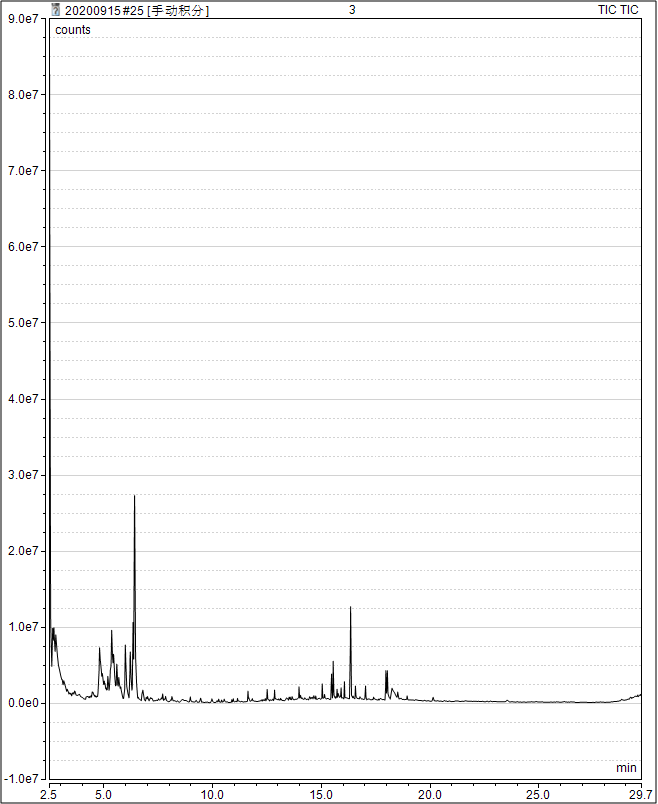

Supplement: S3 Fig — (TIF) [file pone.0276044.s003.tif]

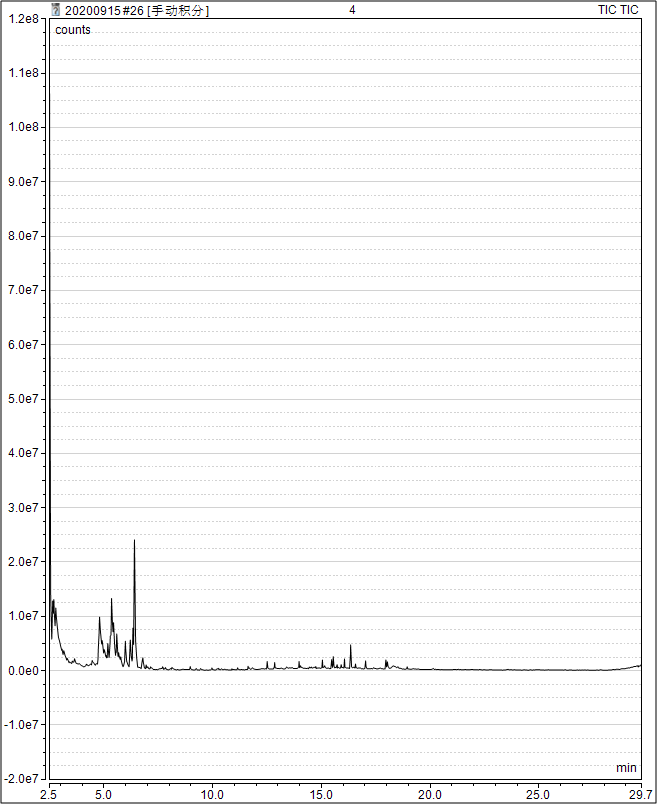

Supplement: S4 Fig — (TIF) [file pone.0276044.s004.tif]

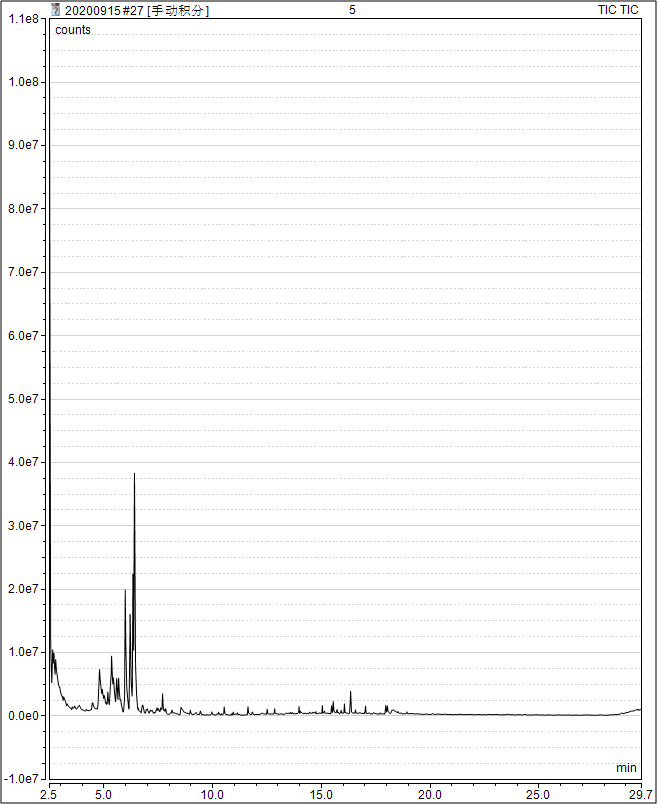

Supplement: S5 Fig — (TIF) [file pone.0276044.s005.tif]

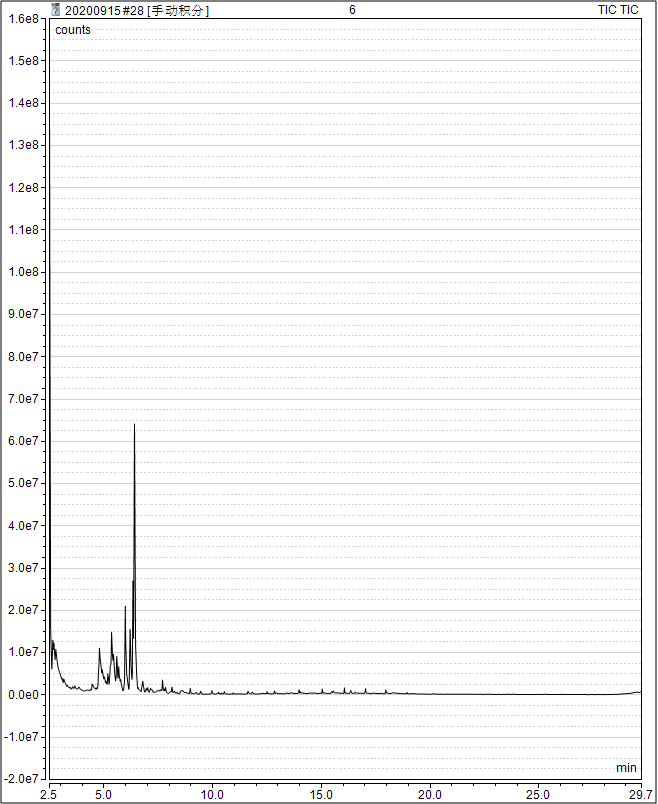

Supplement: S6 Fig — (TIF) [file pone.0276044.s006.tif]

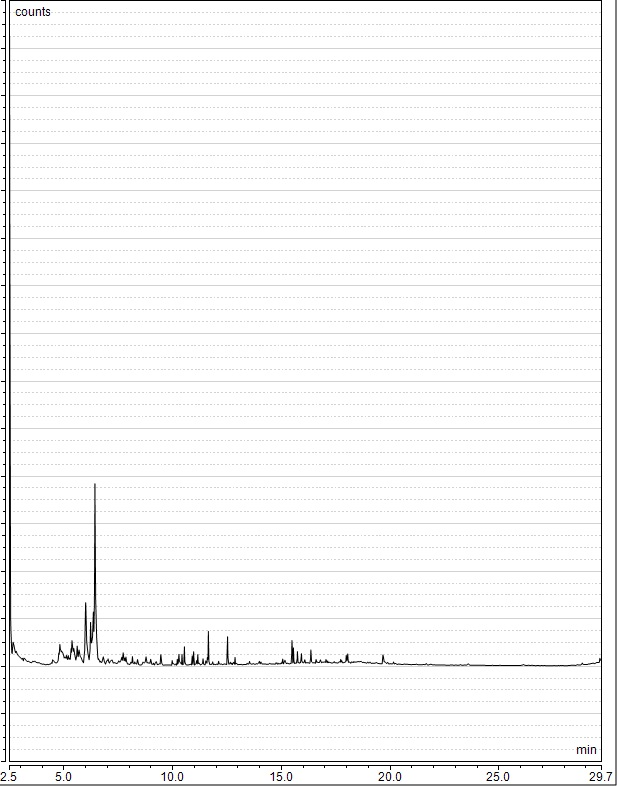

Supplement: S7 Fig — (TIF) [file pone.0276044.s007.tif]

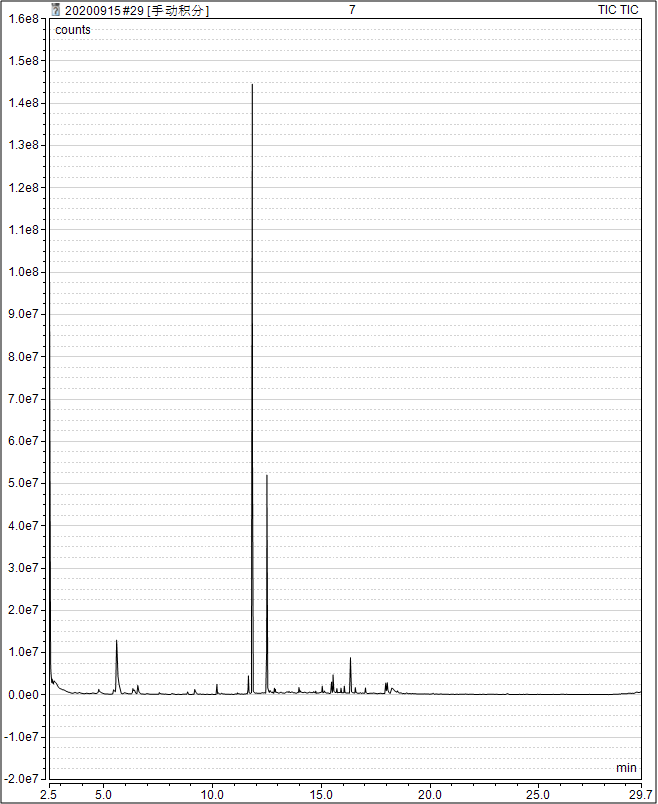

Supplement: S8 Fig — (TIF) [file pone.0276044.s008.tif]

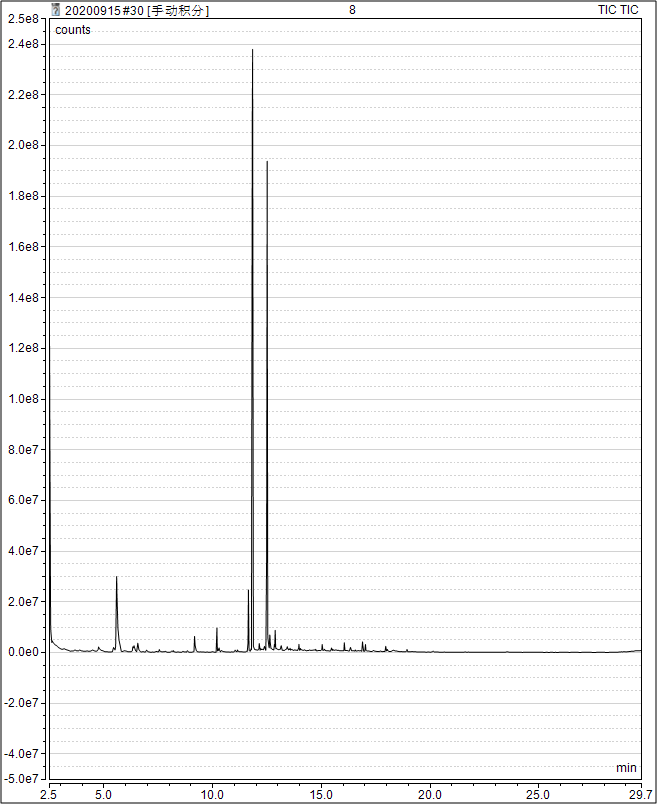

Supplement: S9 Fig — (TIF) [file pone.0276044.s009.tif]

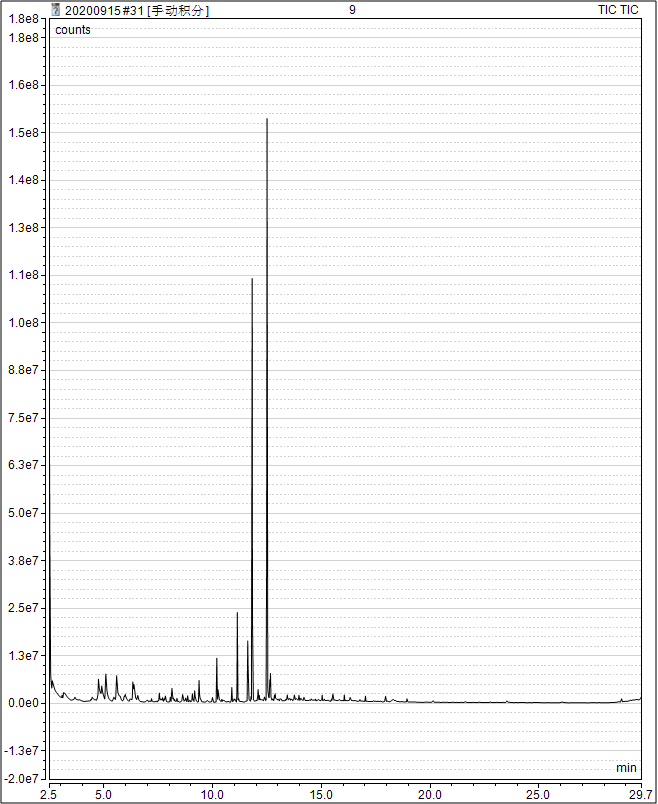

Supplement: S10 Fig — (TIF) [file pone.0276044.s010.tif]

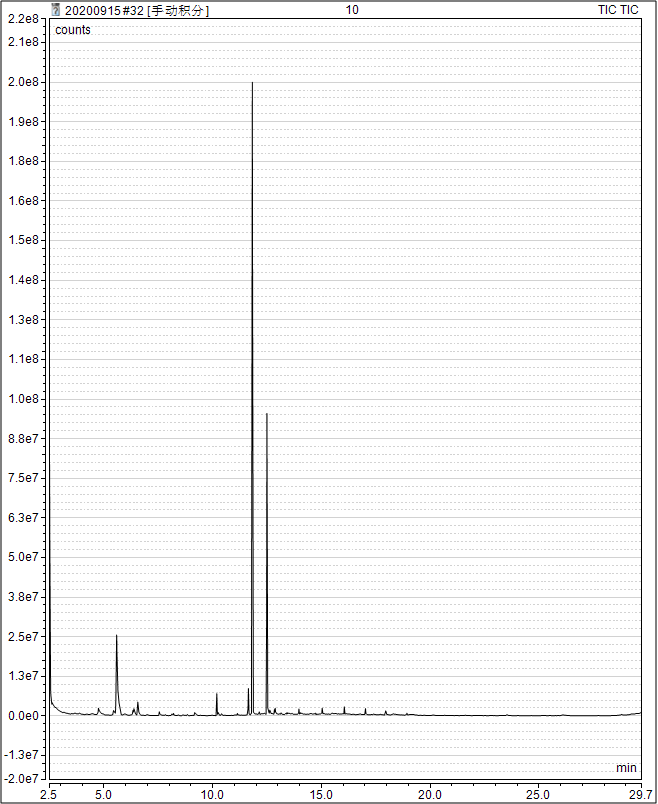

Supplement: S11 Fig — (TIF) [file pone.0276044.s011.tif]

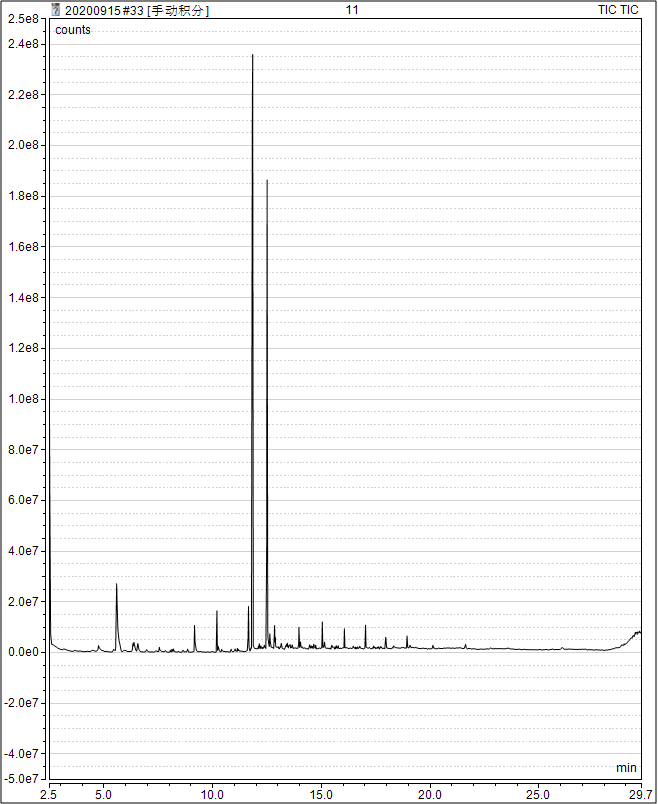

Supplement: S12 Fig — (TIF) [file pone.0276044.s012.tif]

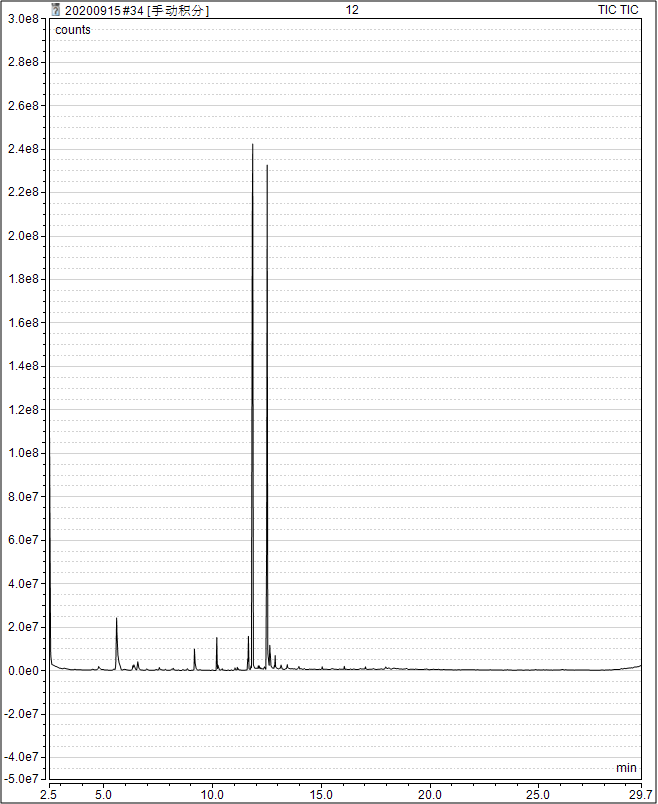

Supplement: S13 Fig — (TIF) [file pone.0276044.s013.tif]

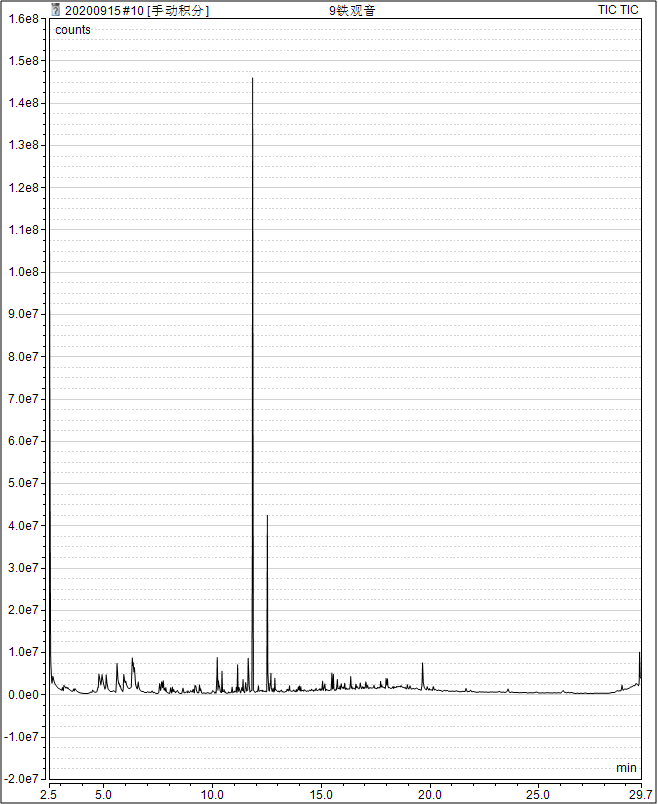

Supplement: S14 Fig — (TIF) [file pone.0276044.s014.tif]

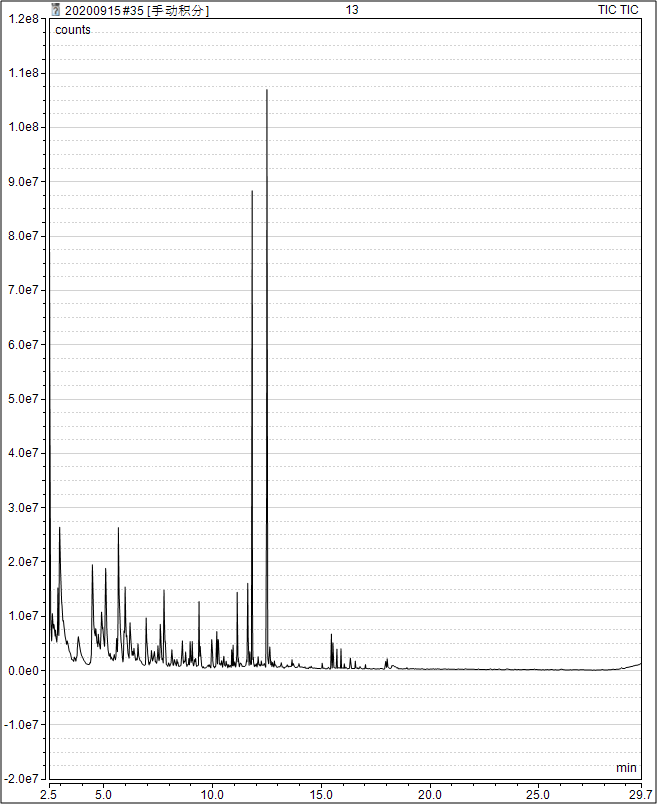

Supplement: S15 Fig — (TIF) [file pone.0276044.s015.tif]

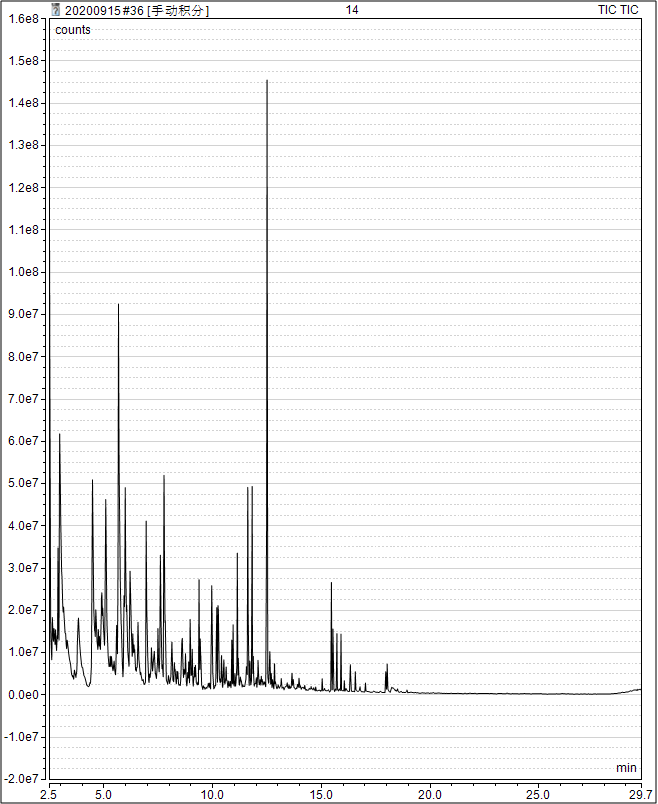

Supplement: S16 Fig — (TIF) [file pone.0276044.s016.tif]

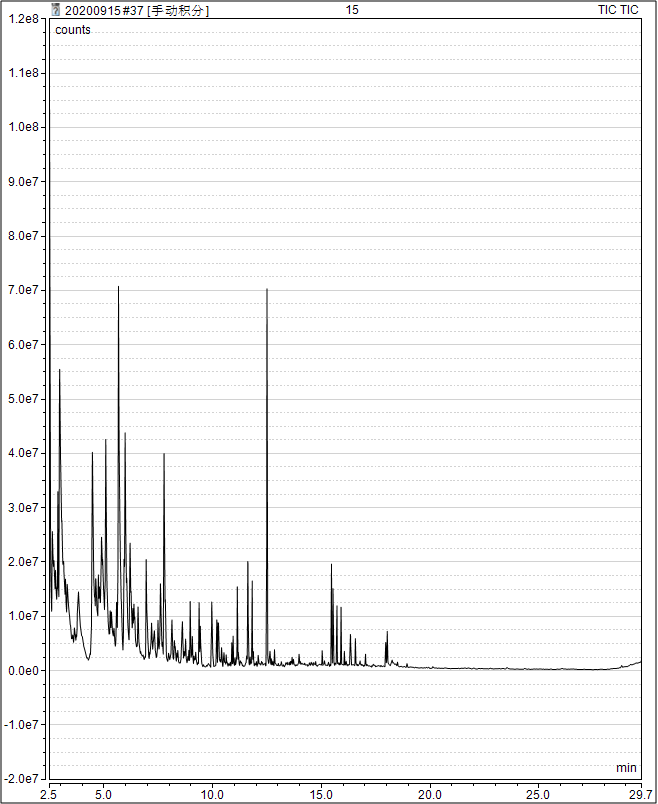

Supplement: S17 Fig — (TIF) [file pone.0276044.s017.tif]

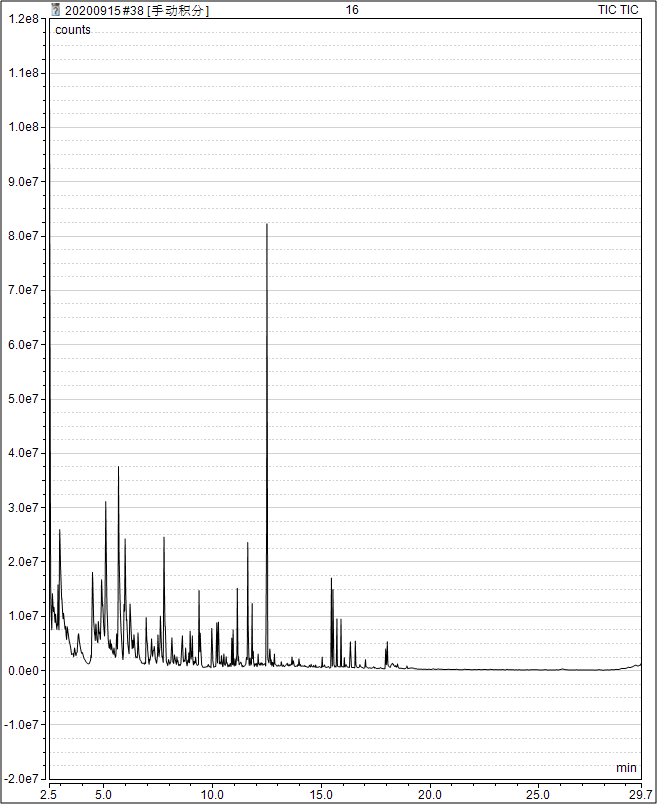

Supplement: S18 Fig — (TIF) [file pone.0276044.s018.tif]

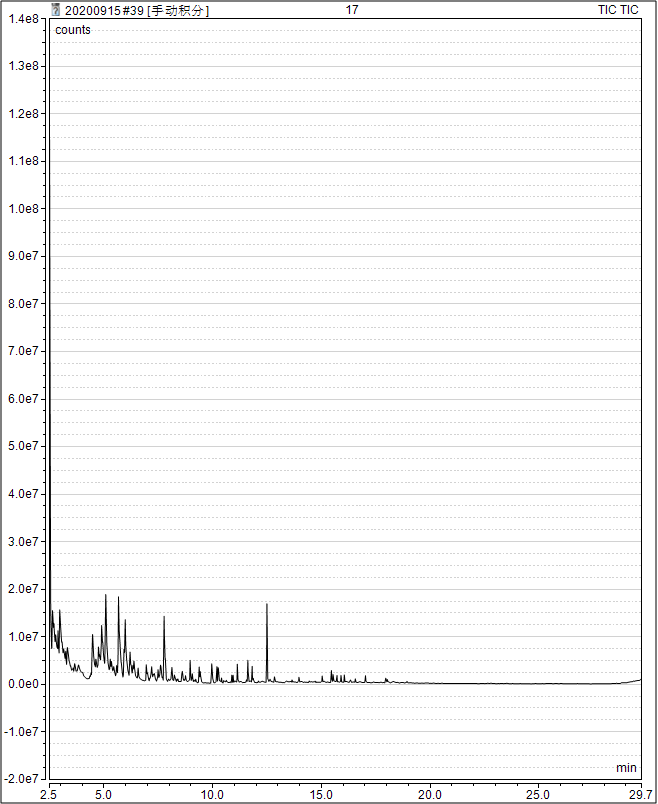

Supplement: S19 Fig — (TIF) [file pone.0276044.s019.tif]

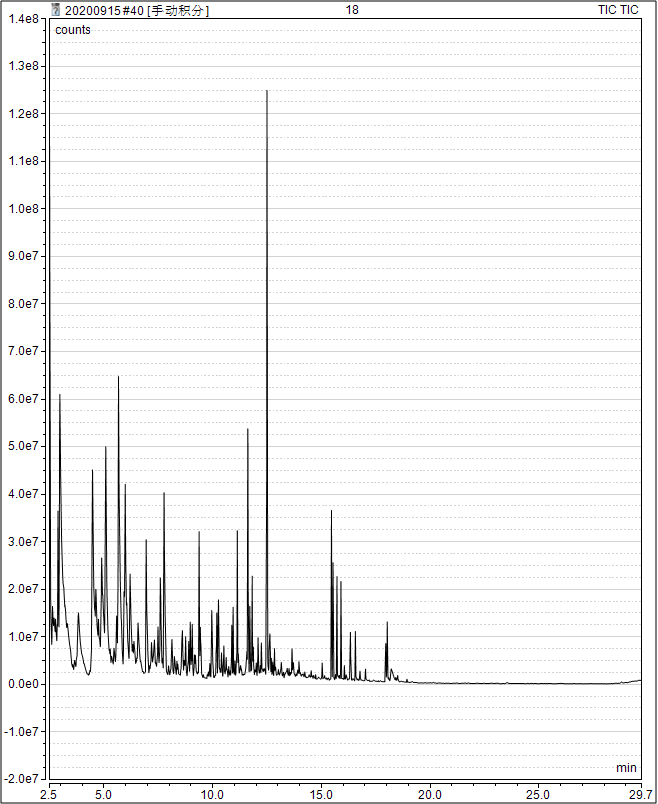

Supplement: S20 Fig — (TIF) [file pone.0276044.s020.tif]

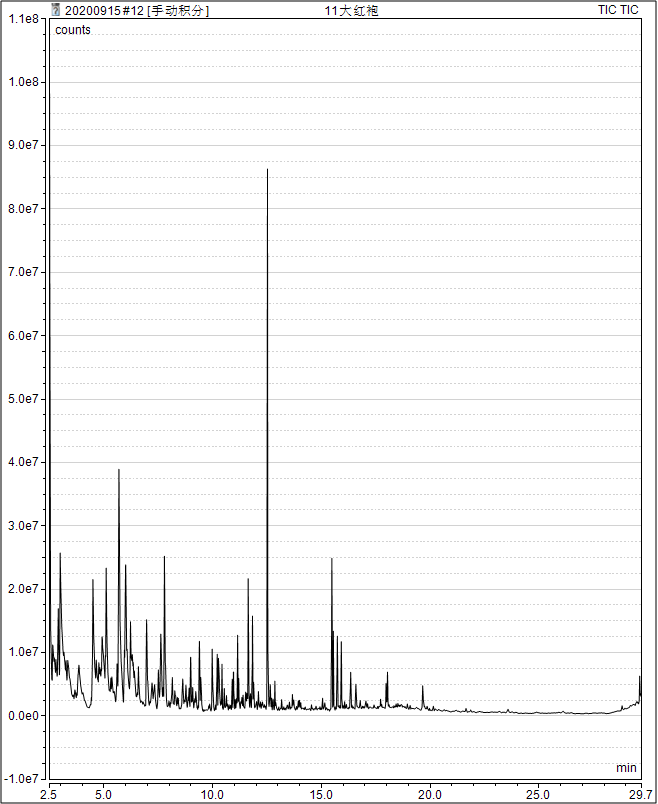

Supplement: S21 Fig — (TIF) [file pone.0276044.s021.tif]
